# Targeting the *α*4*β*2- and *α*7-Subtypes of Nicotinic Acetylcholine Receptors for Smoking Cessation Medication Development

**Published:** 2019-04-15

**Authors:** Lakshmi Ramachandran Nair, Xiu Liu

**Affiliations:** Department of Pathology, University of Mississippi Medical Center, Jackson, MS 39216, USA

**Keywords:** Conditioned stimuli (cues), Nicotinic acetylcholine receptors (nAChRs), Reinforcing actions, Relapse, Self-administration

## Abstract

Nicotine exerts its reinforcing actions via activating the nicotinic acetylcholine receptors (nAChRs). Among an increasing number of nAChR subtypes, the α4β2 and α7 nAChRs are the two major ones, accounting for about 95% of the whole nAChR population in brain. Research findings from our own laboratory, together with other reports in the field, suggest critical and differential involvement of the α4β2 and α7 nAChRs in the process of nicotine dependence and tobacco addiction. Specifically, rat models of nicotine consumption and cue-induced relapse were used to examine the effects of selective antagonism of these two nAChR subtypes on the primary reinforcement of nicotine and the conditioned reinforcing actions of nicotine-associated environmental stimuli (cues). Results demonstrated that blockade of the α4β2 but not α7 subtype effectively reduced nicotine intake, whereas α7 but not α4β2 nAChR blockade reversed cue-triggered nicotine relapse behavior. These findings lend support for the continued effort to develop cholinergic agents aiming at the α4β2 nAChRs for reducing or stopping smoking. However, it is suggested that manipulation of α7 nAChR activity would be a promising target for preventing smoking relapse triggered by exposure to environmental cues.

## Introduction

Tobacco-related diseases are a major problem in many perspectives from human health to social economics [1]. For example, in the United States tobacco smoking becomes a leading preventable cause of premature death. Every year, tobacco smoking results in the loss of 450,000 lives and economic cost of $289 to $333 billion [2]. Currently, approximately 42.1 million American adults are smokers, representing about 18.1% of the population [3]. Although almost all smokers want to quit smoking and make attempts, up to 97% of them relapse to tobacco smoking [3–6]. The high relapse rates of tobacco smoking present a formidable challenge for the success of smoking cessation efforts including currently available pharmacotherapies (e.g., nicotine replacements, bupropion, and varenicline).

Nicotinic acetylcholine receptors (nAChRs) mediate the pharmacological (including its reinforcing) actions of nicotine. These receptors are ion channels composed of five subunits. There are twelve nAChR subunits: nine α-subunits (α2-α10) and three β-subunits (β2-β4). These subunits assemble the nAChRs into either heteromeric (α- and β-subunits) or homomeric (α-subunit only) combinations [7–10]. Increasing number of subtypes of the nAChRs has been and will be identified, among which the heteromeric α4β2- and homomeric α7-containing receptors are the most abundant and widespread subtypes, comprising about 95% of total nAChRs in the brain [7,8,11–14]. These two subtypes show differences in their localization, density, and functional characteristics (e.g., kinetics of activation, desensitization, and recovery from desensitization, and Ca^2+^ permeability) [9,15–18]. Many studies including our own research have demonstrated a critical role of the α4β2 nAChRs in mediating the primary reinforcing actions of nicotine [19–23], while, in contrast, a lack of a clear role of the α7 nAChRs in the nicotine reinforcement [24, 25–31].

Increasing clinical observations and laboratory animal studies have demonstrated the conditioned incentive properties of drug-associated environmental stimuli (cues) [32–37]. In smokers, the environmental cues related to smoking behavior including both distally situational contexts and proximal sensory cues such as the visual and olfactory stimuli associated with each puff elicit subjective states that can trigger smoking and nicotine-seeking behavior [32,33,38–49]. In animal research, the ability of nicotine-related cues to reinstate nicotine-seeking behavior has been well documented [31,37,50–59]. A great interest has focused on the investigation of neurobiological mechanisms underlying the conditioned motivational effects of nicotine cues [36 for a recent review].

Our animal research work over the past decade or so has demonstrated the role of nicotinic neurotransmission in the mediation of the conditioned motivational effects of nicotine-associated cues. The method used for testing the behavior motivational effects of nicotine cues and other relapse risk factors (e.g., stress or drug priming) was the response-reinstatement tests, which has been validated to be an animal model of relapse [60–62]. Using this testing procedure, we for the first time found that a nonselective nAChR antagonist mecamylamine effectively reversed the cue-induced reinstatement of nicotine-seeking behavior [63]. And furthermore, our recent work has demonstrated that the α7 but not α4β2 nAChRs mediate the cue-induced reinstatement of nicotine-seeking behavior [31].

## Experimental Procedures for Testing Nicotine Consumption and Relapse

Rats were used for testing nicotine intake and relapse behavior. After implantation of an indwelling intravenous catheter, the animals were trained to self-administer nicotine in daily one-hour sessions in the standard operant conditioning chambers. In the sessions, once the rats reached a fixed-ratio 5 schedule requirement of responses on the active lever, an infusion of nicotine was delivered. To establish a nicotine-conditioned cue, each nicotine infusion was paired with presentation of a sensory stimulus. For testing cue-triggered relapse behavior, extinction sessions were performed after completion of the self-administration and conditioning training. In these sessions, responses on the lever produced no programed outcomes. After responding was extinguished, the response-reinstatement test sessions were conducted where lever-pressing responses led to the cue presentations while without the delivery of nicotine [54–55,63]. The antagonists of the nAChRs were administered to the rats prior to the self-administration and the reinstatement test sessions [31,63]. The antagonists included a nonselective antagonist mecamylamine, a α4β2-selective antagonist dihydro-β-erythroidine (DhβE), and a α7-selective antagonist methyllycaconitine (MLA).

## Distinct Roles of the α4β2 and α7 nAChRs in the Reinforcement by Nicotine versus Conditioned Motivation by Nicotine Cues

Ample evidence obtained from both human and animal studies has demonstrated a clear role of the α4β2 nAChRs in mediating the primary reinforcement by nicotine [19–23]. In contrast, however, it is not quite clear whether the α7 nAChRs play a role in nicotine primary reinforcement [24,25–31]. For example, in one study MLA did not interfere with nicotine self-administration [24], whereas, in another report MLA produced a suppressant effect [64]. Conditioned place preference studies also excluded a possible role for α7 nAChRs in the mediation of nicotine reward. For example, mice that were either treated with MLA or deficient in α7 nAChRs developed nicotine-induced conditioned place preference at a level similar to their control counterparts [28,65]. Interestingly, Brunzell and McIntosh [29] found that the α7 nAChR-selective antagonist α-conotoxin ArlB [VIIL, VI6D], when microinjected into rat nucleus accumbens shell and anterior cingulate cortex, significantly increased nicotine self-administration behavior under a progressive-ratio schedule of reinforcement. Of significance is our recent research showing that MLA did not change the self-administration of nicotine [31], indicating that activation of the α7 nAChRs is not required for the reinforcement by nicotine. In summary, activation of α7 nAChRs is proposed to play little, if any at all, role in the mediation of nicotine primary reinforcement.

In the response-reinstatement tests, response-contingent presentation of the nicotine-conditioned cues triggered the recovery of lever-press responding after extinction. Such an effect was specific for nicotine-seeking behavior in that responses on the inactive lever remained unchanged, indicating the unlikelihood of a result of nonspecific behavioral arousal. The conditioned incentive properties of nicotine cues have been very well documented in literature including our own series of studies over the last decade or so [31,37,50–59]. These results obtained from animal research lend support for clinical observations that smoking-related cues enhance desire to smoke [32,33,38–49]. Together, these findings suggest that re-exposure to environmental stimuli previously associated with nicotine intake can play an important role in relapse to tobacco smoking in abstinent smokers.

The nonselective nAChR antagonist mecamylamine effectively reversed the recovery of nicotine-seeking behavior triggered by nicotine cue presentation. This finding demonstrates the requirement of neurotransmission via the nAChRs for the expression of cue-triggered relapse to smoking behavior. It is consistent with clinical observations. For example, mecamylamine was reported to decrease the likeness for intravenously infused nicotine in smokers [66], craving for smoking [67], and satisfaction following smoking [68–70].

In contrast to the role of the α4β2 nAChRs in mediating nicotine reinforcement, our studies showed that blockade of these receptors by DhβE pre-treatment did not interfere with the cue-induced reinstatement of nicotine-seeking responses [31]. The doses used should be sufficient to antagonize the receptors because such a dose range has often been used in the literature, including selfadministration studies [24,71,72] and our own previous study showing its suppressant effect on nicotine-enhanced lever-pressing behavior in response to the presentation of a reinforcing stimulus [73]. These results are consistent with other studies. For example, varenicline, a partial agonist at α4β2 nAChRs, had no effect on the cue-induced reinstatement of nicotine seeking assessed using similar extinction-reinstatement procedures in rodents [74,75] and did not change cue-induced craving in smokers [76]. However, blockade of the α4β2 nAChRs by (DhβE did not change the cue-triggered recovery of nicotine-seeking behavior. That indicates the lack of a role of the neurotransmission via the α4β2 nAChRs in mediating the conditioned incentive motivation by nicotine cue exposure.

It is interesting to note that blockade of the α7 nAChRs by MLA dose-dependently reduced the cue-triggered recovery of nicotine-seeking behavior. It demonstrates the requirement of the activation of the α7 nAChRs mediating the conditioned incentive motivation by exposure to nicotine-conditioned environmental cues. Since neither did MLA alter the recovery of cue-triggered food-seeking behavior nor changed the enhancing effect of nicotine on other intrinsically reinforcing sensory stimulus [73], MLA acted specifically at the nicotine cue without interference with general locomotor activity, arousal state, the motivation to earn rewards, and operant goal-directed behavior. Therefore, the specific inhibitory effect of MLA on the cue-induced resumption of nicotine seeking suggests that activation of α7 nAChRs is necessary for the expression of conditioned incentive motivation induced by nicotine-related cues. Our recent studies (not yet published) further demonstrated that α-conotoxin ArlB [VIIL, VI6D] microinjected into the nucleus accumbens but not ventral tegmental area effectively blocked the cue-triggered reinstatement of nicotine-seeking responses. The finding indicates that the nucleus accumbens is a critical neuroanatomical substrate for the α7 nAChRs to mediate the behavioral effect of nicotine cues.

## Implications for Development of Smoking Cessation Medications

Converging experimental evidence suggests that the α4β2 and α7 nAChRs play differential roles in mediating the reinforcing actions of nicotine versus the conditioned incentive properties of nicotine cues. The α4β2 nAChRs participate in nicotine primary reinforcement but not conditioned reinforcement induced by nicotine cues, whereas α7 nAChRs do the opposite. The differential involvement of these two nAChR subtypes indicates a dissociation of the neurobiological mechanisms that underlie the primary reinforcing actions of nicotine and secondary reinforcement induced by nicotine cues. High level of α7 nAChRs is expressed in the cortico mesolimbic circuits, including profrontal cortex, ventral tegmental area and nucleus accumbens as well as the hippocampus and hypothalamus [18,77–79]. These receptors may play an essential role in the modulation of dopamine rewarding pathways in that agonists produce cognition enhancement [80]. That may underlie the role of α7 nAChRs in mediating the conditioned incentive properties of nicotine cues. Interestingly, similar dissociations were also demonstrated with other drugs of abuse. For example, pharmacological antagonism of opioid receptors decreased cue-induced resumption of nicotine seeking but did not alter nicotine intake [81]. Inhibition of nitric oxide synthesis reduced cue-triggered recovery of alcohol seeking but not the consumption of alcohol [82]. Blockade of orphan sigma-1 receptors decreased cue-induced recovery of cocaine-seeking responses, produced no effect on cocaine intake [83]. In summary, the reinforcement of nicotine and the conditioned motivation by nicotine cues involve distinct neurobiological mechanisms.

The research work reviewed above supports the continued effort to develop nicotinic agents aiming at the α4β2 nAChRs for reducing and eventually stopping nicotine consumption and tobacco smoking. The α4β2 targeted medications have found increasing clinical use although their efficacy is not yet quite satisfactory [84–86]. However, the lack of involvement of the α4β2 nAChRs in nicotine cue effect might help explain the inability of currently available smoking cessation medications (nicotine replacement, bupropion, and varenicline) to suppress cue-reactivity in abstinent subjects [74,75,87–90] because these medications are full (nicotine) or partial (varenicline) agonist or antagonist (one of bupropion’s actions) at the α4β2 nAChRs. In light of the fact that cholinergic neurotransmission via the α7 subtype of nAChRs plays a critical role in mediating the conditioned incentive properties of nicotine cues, it is suggested that developing cholinergic agents aiming at the α7 nAChRs may prove to be a good strategy to prevent smoking relapse triggered by exposure to environmental cues.

These preclinical research results would have significant implications for developing medication strategies to prevent relapse in abstinent smokers. To our knowledge, however, there has been no clinical trial performed to test the potential of α7 nAChR antagonists for smoking relapse prevention. Although there have been many studies to examine characteristics of the brain nAChRs [7–18], it is lack of direct comparison of these receptors such as the α4β2 and α7 subtypes among different species, e.g., rodents versus humans. These facts call for more research effort to address the issues and acknowledge the caveats for clinical tests, if any in the future.
